# Risk of anxiety disorder following ankylosing spondylitis, 2012-2023: a nationwide cohort study in South Korea

**DOI:** 10.3389/fpsyt.2026.1686890

**Published:** 2026-02-13

**Authors:** Seung Won Lee, Yejin Lee, Chaeyoon Kang, Haerim Cho, Jin Hoon Park, Jae June Dong, Habtamu Milkias Wolde, Won-Suk Shin, Hohyun Jung, Youngoh Bae

**Affiliations:** 1 Department of Precision Medicine, Sungkyunkwan University School of Medicine, Suwon, Republic of Korea; 2 Department of Family Medicine, Kangbuk Samsung Hospital, Sungkyunkwan University School of Medicine, Seoul, Republic of Korea; 3 Dongguk University College of Medicine, Gyeongju, Republic of Korea; 4 Sungkyunkwan University School of Medicine, Seoul, Republic of Korea; 5 Department of Neurological Surgery, Asan Medical Center, University of Ulsan College of Medicine, Seoul, Republic of Korea; 6 Department of Physiology, School of Medicine, CHA University, Pocheon-si, Republic of Korea; 7 Department of Statistics, Sungshin Women’s University, Seoul, Republic of Korea; 8 Data Science Center, Sungshin Women’s University, Seoul, Republic of Korea; 9 Department of Neurosurgery, Korean Armed Forces Capital Hospital, Seoul, Republic of Korea

**Keywords:** ankylosing spondylitis, anxiety disorder, cohort study, risk factors, South Korea

## Abstract

**Introduction:**

Ankylosing spondylitis (AS) is a chronic inflammatory rheumatic disease associated with impaired quality of life and psychiatric comorbidities. Although depression has been widely studied in AS, the risk of anxiety disorders remains unclear. This study examined the long-term risk of anxiety disorders in patients with AS using a nationwide Korean cohort.

**Methods:**

Using the Korean National Health Insurance Service database (2012–2023), we identified patients newly diagnosed with ankylosing spondylitis (ICD-10 code M45) after applying a 3-year washout period to minimize inclusion of pre-existing cases. Patients with a prior history of anxiety disorders were excluded. Each case was propensity score–matched at a 1:10 ratio with controls by age, sex, and index year. The primary outcome was incident anxiety disorder (ICD-10 codes F40–F41). Adjusted hazard ratios (aHRs) and 95% confidence intervals (CIs) were estimated using Cox proportional hazards models.

**Results:**

We analyzed 2,762 patients with AS and 27,620 controls over a mean follow-up of 4.02 years. The incidence rate of anxiety disorder was 27.38 per 1,000 person-years in AS and 18.91 in controls (IRR, 1.45; 95% CI, 1.28–1.63). AS was associated with a 40% higher risk of anxiety disorder (aHR, 1.40; 95% CI, 1.14–1.73), with the strongest association in females <60 years (aHR, 1.87; 95% CI, 1.52–2.30).

**Conclusions:**

AS increases the risk of anxiety disorders, particularly in younger females. Therefore, early psychiatric screening should be considered during AS management.

## 1 Introduction

Ankylosing spondylitis (AS) is a common chronic inflammatory rheumatic disease that primarily affects the axial skeleton, including the spine and sacroiliac joints (1). The global prevalence of AS varies across continents, ranging from 7.4 to 31.9 per 10,000 individuals (1). In addition to characteristic manifestations such as inflammatory back pain, stiffness, and reduced spinal mobility, AS is frequently accompanied by various systemic conditions, including uveitis, psoriasis, inflammatory bowel disease, and cardiovascular disorders (2). These physical manifestations impose substantial socioeconomic strain through reduced quality of life, loss of productivity, and increased healthcare costs, with direct medical expenses for AS reported to exceed those for chronic low back pain (3, 4).

There is growing recognition that the impact of AS extends beyond physical symptoms to mental health. Patients with AS have been reported to exhibit an elevated risk for various psychiatric disorders, including depressive disorders, anxiety disorders, and sleep disturbances (5). In a nationwide cohort study conducted in Sweden, the standardized incidence ratio of anxiety disorders in patients with AS was higher than that of depressive disorders and other psychiatric subtypes (6). Furthermore, the risk of anxiety disorders in patients with AS exceeded that observed in patients with systemic lupus erythematosus (SLE) or rheumatoid arthritis (RA) (6). These findings suggest that AS may be an independent risk factor for psychiatric disorders, particularly anxiety disorders, underscoring the importance of early recognition and timely intervention.

Nevertheless, large-scale population-based studies with long-term follow-up on the risk of anxiety disorders in patients with AS remain scarce. Most previous studies have primarily focused on depressive disorders, with limited investigations on anxiety disorders (5). However, a nationwide population-based study from Taiwan reported that patients with AS had a significantly higher risk of developing anxiety disorders compared to controls, with an adjusted hazard ratio of 1.85 (95% confidence interval [CI], 1.37–2.49), indicating a potential association between AS and anxiety disorders (7).

In patients with AS, anxiety disorders are closely associated with higher pain intensity (8), lower quality of life (9), and higher disease activity (10, 11). Therefore, early screening and proactive management are critical for improving patient outcomes.

Using nationwide health insurance data from Korea, this study aimed to evaluate the long-term risk of anxiety disorders following a diagnosis of AS. Using a retrospective cohort design with a propensity score–matched control group, the study sought to examine the association between AS and anxiety disorders and identify risk factors influencing their occurrence. These findings are expected to inform the development of personalized mental health interventions for the management of patients with AS.

## 2 Methods

### 2.1 Data source

The Korean National Health Insurance Service (NHIS) is a government-administered mandatory social health insurance program that covers nearly the entire population (approximately 97%) (12). The NHIS manages all medical expenditures involving beneficiaries, healthcare providers, and the government. Owing to the unique personal identification numbers assigned at birth, medical records in Korea are neither duplicated nor omitted. The NHIS database contains nearly all medical information, including diagnostic codes, surgical procedures, and prescribed medications. The dataset used in this study included demographic variables, such as age, sex, and income quintile, as well as primary and secondary diagnostic information.

In addition, a nationwide health-screening program is offered to all citizens aged ≥20 years. Depending on individual eligibility, participants undergo health examinations annually or biennially. According to 2013 NHIS statistics, 72.1% of eligible beneficiaries receive a general health examination (13).

The need for ethical approval for this study was waived by the Institutional Review Board of Sungshin Women’s University (SSWUIRB-2024-039). As all NHIS data were anonymized to prevent the identification of individuals, the requirement for informed consent was waived.

### 2.2 Study population

This retrospective cohort study covered the period from January 2012 to December 2023. The AS cohort was defined as individuals who received an ICD-10 code of M45 for either a primary or secondary diagnosis at least twice between 2015 and 2023, with the second diagnosis set as the index date.

Since 2009, the NHIS has included AS in the Rare Intractable Disease (RID) registration program. Under this program, ICD-10 code M45 is assigned to patients who meet the modified New York criteria for AS (14), which include (1) low back pain and stiffness lasting ≥3 months that improves with exercise but not with rest, (2) limitation of lumbar spine motion in both the sagittal and coronal planes, and (3) reduced range of thoracic expansion after adjustment for sex and age. The radiological criterion is bilateral sacroiliitis grade ≥II or unilateral sacroiliitis grade ≥III. A diagnosis of AS is made when at least two of these criteria, including radiological criteria, are met.

For RID registration, all patients must be diagnosed by a physician according to the standardized criteria provided by the NHIS, and all diagnoses must undergo an additional review process to confirm eligibility. This procedure ensures the diagnostic validity of AS cases registered in the RID program (15).

To ensure inclusion of only newly diagnosed AS cases, a 3-year washout period (2012–2014) was applied to minimize the likelihood of including long-standing but previously undiagnosed patients. In Korea, AS is registered under the Rare Intractable Disease (RID) program, for which strict clinical and radiologic criteria with additional review are required, thereby enhancing diagnostic validity. Furthermore, at least two separate records of the AS diagnosis code (M45) were required for cohort entry, reducing the risk of misclassification. Although this approach cannot fully distinguish incident from delayed diagnoses, the likelihood that cases identified from 2015 onward represent newly diagnosed AS is substantially increased.The exclusion criteria for the AS cohort were as follows: (1) diagnosis of AS or any major comorbidity related to the study outcomes during the washout period (**Supplementary Table S1**); (2) absence of health screening records within 3 years prior to the index date; (3) age <20 or ≥80 years at the index date; and (4) diagnosis of anxiety disorder within 3 years prior to the index date.

The control cohort was selected from individuals who participated in the National Health Screening Program (NHSP) between 2012 and 2023 and had no diagnosis of AS. The same exclusion criteria were applied. Controls were matched to cases in a 1:10 ratio by sex, age, and year of health screening using propensity score matching. Covariates included smoking status, alcohol consumption, body mass index (BMI), total cholesterol, systolic blood pressure, diastolic blood pressure, fasting blood sugar (FBS), and income level. Missing data for these covariates were handled using a single imputation with predictive mean matching. The index date for the controls was set to match that for the matched cases.

### 2.3 Main outcome

Psychiatric ICD-10 codes in South Korea are determined by board-certified psychiatrists in accordance with DSM-5 diagnostic standards. To ensure diagnostic rigor and minimize the risk of misclassification, the definition of anxiety disorders was restricted to ICD-10 codes F40–F41. Related conditions that do not primarily represent anxiety disorders, such as adjustment disorders and somatoform disorders, were excluded (**Supplementary Table S1**). The primary outcome was defined as a newly diagnosed anxiety disorder, which was operationalized as at least two separate records of ICD-10 codes F40 or F41, with a minimum interval of six months between the records to confirm diagnostic validity. Participants were followed from the index date, which was defined as the date of the first qualifying diagnosis, until the earliest occurrence of anxiety disorder, death, or December 31, 2023.

### 2.4 Covariates

The covariates included sex, age, smoking status, alcohol consumption, height, weight, BMI, total cholesterol, systolic blood pressure, diastolic blood pressure, FBS, and income level. Values closest to the health-screening date were used. Height and weight were analyzed as continuous variables, and all other covariates were analyzed as categorical variables.

Health screenings were conducted at NHIS-certified medical institutions under regular quality control. Blood pressure was measured by trained clinicians after participants had rested for at least 5 min, with the arm positioned at the heart level. Blood samples were collected after overnight fasting, and total cholesterol and FBS levels were measured using standardized protocols. BMI (kg/m^2^) was categorized as underweight (<18.5), normal (18.5–24.9), or obese (≥25). Total cholesterol level (mg/dL) was classified as normal (<200), borderline (200–239), or hypercholesterolemic (≥240). Systolic blood pressure (mmHg) was categorized as normal (<120), prehypertensive (120–139), or hypertensive (≥140), whereas diastolic blood pressure was categorized as normal (<80), prehypertensive (80–89), or hypertensive (≥90). FBS levels (mg/dL) were defined as normal (<100), impaired (100–125), or hyperglycemic (≥126). Income level was dichotomized into ≥50th and <50th percentiles of the general population.

### 2.5 Statistical analysis

Differences in baseline characteristics between cohorts were assessed using Student’s t-test for continuous variables and Fisher’s exact test or Pearson’s χ² test for categorical variables. Standardized differences were also calculated, with values <0.1 considered negligible. Incidence rates (IRs) of anxiety disorders were calculated per 1,000 person-years, and IR ratios (IRRs) were computed to compare cohorts. Kaplan–Meier curve analysis and log-rank test were employed to compare the time-to-event occurrence of anxiety disorders. The association between AS and anxiety disorders was estimated using multivariable Cox proportional hazards models, adjusting for sex, age, smoking status, alcohol consumption, BMI, total cholesterol, and income level. Subgroup analyses were performed according to age and sex. All statistical analyses were conducted using R software (version 4.4.1), and two-sided p-values <0.05 were considered statistically significant.

## 3 Results

**Figure 1:**
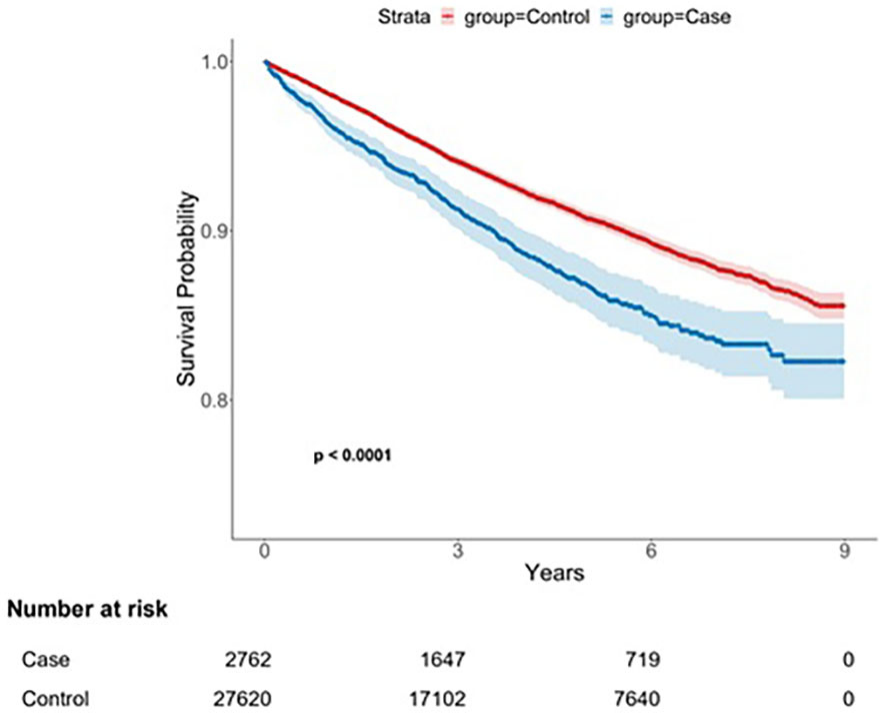
Flowchart for case and control group selection. AS, ankylosing spondylitis.

A total of 2,762 patients with AS and 27,620 propensity score–matched controls were included in the analysis (**Figure 1**). In the AS cohort, 2,069 individuals (74.9%) were aged < 60 years and 1,616 (58.5%) were male. The mean follow-up duration was 4.02 years (standard deviation [SD], 2.56) in the AS cohort and 4.17 years (SD, 2.55) in the control cohort. No statistically significant differences in baseline demographic characteristics were observed between the cohorts (**Table 1**).

**Table 1:** Baseline characteristics of patients with ankylosing spondylitis (AS) and matched controls.

Variables	Categories	AS case group (n = 2,762) (%)	Control group (n = 27,620) (%)	Standardized difference	*P*–value
Age (years)	20–29	373 (13.50)	3730 (13.50)	0.00	1.00
	30–39	562 (20.35)	5620 (20.35)		
	40–49	551 (19.95)	5510 (19.95)		
	50–59	583 (21.11)	5830 (21.11)		
	60–69	441 (15.97)	4410 (15.97)		
	≥70	252 (9.12)	2520 (9.12)		
Sex	Male	1616 (58.51)	16160 (58.51)	0.00	1.00
	Female	1146 (41.49)	11460 (41.49)		
Smoking status	Current	549 (19.90)	5366 (19.44)	0.02	0.77
	Never	1627 (58.97)	16467 (59.65)		
	Former	583 (21.13)	5774 (20.91)		
	Missing	3 (0.11)	13 (0.05)		
Frequency of alcohol consumption (per week)	0	831 (40.03)	8454 (39.11)	0.02	0.71
	1–2	973 (46.87)	10284 (47.57)		
	≥3	272 (13.10)	2879 (13.32)		
Weight (kg, mean ± SD)		66.61 ± 12.72	66.39 ± 12.73	0.02	0.38
Height (cm, mean ± SD)		164.42 ± 9.66	164.43 ± 9.63	0.00	0.94
BMI (kg/m^2^)	<18.5	84 (3.04)	666 (2.41)	0.04	0.10
	18.5 to <25	1552 (56.23)	15802 (57.23)		
	≥25	1124 (40.72)	11145 (40.36)		
Total cholesterol (mg/dL)	<200	1090 (60.15)	10245 (59.23)	0.03	0.46
	≥200	722 (39.85)	7053 (40.77)		
Systolic blood pressure (mmHg)	<120	1020 (37.12)	10435 (37.97)	0.02	0.59
	120 to <140	1351 (49.16)	13427 (48.85)		
	≥140	377 (13.72)	3623 (13.18)		
Diastolic blood pressure (mmHg)	<80	1595 (58.04)	16199 (58.94)	0.04	0.22
	80 to <90	879 (31.99)	8815 (32.07)		
	≥90	274 (9.97)	2471 (8.99)		
FBS (mg/dL)	<100	1634 (59.48)	16491 (60.00)	0.02	0.83
	100 to <126	853 (31.05)	8383 (30.50)		
	≥126	260 (9.46)	2611 (9.50)		
Income	Low	1069 (40.37)	10643 (39.96)	0.01	0.69
	High	1579 (59.63)	15993 (60.04)		

BMI, body mass index, FBS, fasting blood sugar.

**Figure 2:**
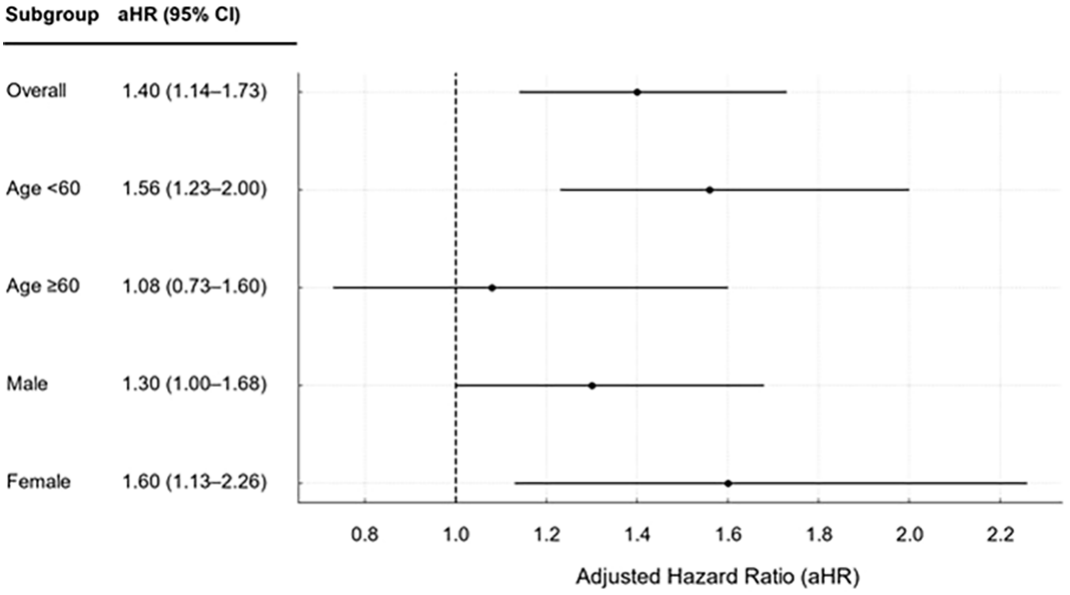
Kaplan–Meier survival curve for anxiety disorder following ankylosing spondylitis diagnosis.

The cumulative incidence of anxiety disorders was significantly different between the AS and control cohorts (log-rank test, p < 0.001; **Figure 2**). Over the entire follow-up period, 304 patients (11.0%) in the AS cohort and 2,178 individuals (7.89%) in the control cohort were diagnosed with anxiety disorders (**Table 2**). The incidence rate (IR) per 1,000 person-years was 27.38 (95% confidence interval [CI], 24.32–30.53) in the AS cohort and 18.91 (95% CI, 18.12–19.71) in the control cohort, resulting in an incidence rate ratio (IRR) of 1.45 (95% CI, 1.28–1.63).

**Table 2:** Crude incidence rates and incidence rate ratios of anxiety disorders in patients with ankylosing spondylitis.

Variables	Categories	Case cohort (n = 2,762)			Reference cohort (n = 27,620)			IRR (95% CI)
		Cases	Person–years	IR per 1000 person–years (95% CI)	Cases	Person–years	IR per 1000 person–years (95% CI)	
All		304	11103.14	27.38 (24.32–30.53)	2178	115149.74	18.91 (18.12–19.71)	1.45 (1.28–1.63)
Age (years)	<60	196	8195.58	23.92 (20.75–27.28)	1218	84610.97	14.40 (13.61–15.21)	1.66 (1.42–1.93)
	≥60	108	2907.57	37.15 (30.72–44.07)	960	30538.78	31.44 (29.49–33.48)	1.18 (0.96–1.45)
Sex	Male	135	6720.31	20.09 (16.81–23.51)	1009	68625.70	14.70 (13.80–15.62)	1.37 (1.14–1.64)
	Female	169	4382.83	38.56 (32.86–44.49)	1169	46524.04	25.13 (23.69–26.57)	1.53 (1.31–1.80)
Sex and Age (years)	Male, <60	92	5295.55	17.37 (13.97–20.96)	626	53721.44	11.65 (10.74–12.58)	1.49 (1.20–1.86)
	Male, ≥60	43	1424.76	30.18 (21.76–39.30)	383	14904.26	25.70 (23.15–28.31)	1.17 (0.86–1.61)
	Female, <60	104	2900.03	35.86 (29.31–42.76)	592	30889.52	19.17 (17.64–20.72)	1.87 (1.52–2.30)
	Female, ≥60	65	1482.80	43.84 (33.72–54.63)	577	15634.52	36.91 (33.90–39.98)	1.19 (0.92–1.53)
Smoking status	Yes	47	2430.81	19.34 (13.99–25.09)	291	23240.08	12.52 (11.10–13.98)	1.54 (1.13–2.10)
	No	201	6431.18	31.25 (27.06–35.61)	1479	67737.28	21.83 (20.73–22.96)	1.43 (1.24–1.66)
	Ex–Smoking	56	2231.39	25.10 (18.82–31.82)	408	24119.39	16.92 (15.30–18.57)	1.48 (1.12–1.96)
Frequency of alcohol consumption (per week)	0	74	2845.25	26.01 (20.38–31.98)	587	31003.88	18.93 (17.42–20.48)	1.37 (1.08–1.75)
	1–2	76	3267.46	23.26 (18.06–28.77)	532	35958.43	14.79 (13.54–16.07)	1.57 (1.24–2.00)
	≥3	22	1007.22	21.84 (12.91–31.77)	169	10492.57	16.11 (13.72–18.58)	1.36 (0.87–2.11)
BMI (kg/m^2^)	<18.5	9	342.68	26.26 (11.67–43.77)	55	2764.80	19.89 (14.83–25.32)	1.32 (0.65–2.67)
	18.5 to <25	185	6242.02	29.64 (25.47–33.96)	1291	66750.20	19.34 (18.29–20.40)	1.53 (1.31–1.79)
	≥25	110	4511.05	24.38 (19.95–29.04)	832	45604.86	18.24 (17.02–19.49)	1.34 (1.10–1.63)
Total cholesterol (mg/dL)	<200	144	5265.34	27.35 (22.98–31.91)	1023	52475.30	19.49 (18.31–20.70)	1.40 (1.18–1.67)
	≥200	90	3511.79	25.63 (20.50–31.04)	681	35783.82	19.03 (17.61–20.48)	1.35 (1.08–1.68)
Income	Low	132	4270.83	30.91 (25.76–36.29)	824	43541.64	18.92 (17.64–20.23)	1.63 (1.36–1.96)
	High	164	6356.32	25.80 (21.87–29.89)	1271	67436.17	18.85 (17.82–19.89)	1.37 (1.16–1.61)

CI, confidence interval, IR, incidence rate, IRR, incidence rate ratio.

In the subgroup analyses by sex and age, the highest IRR was observed in females aged < 60 years (1.87; 95% CI, 1.52–2.30), followed by younger males (1.49; 95% CI, 1.20–1.86), older females (1.19; 95% CI, 0.92–1.53), and older males (1.17; 95% CI, 0.86–1.61). The IRRs of older males and females were not statistically significant. Analyses stratified by smoking status, alcohol consumption, BMI, total cholesterol level, and income level revealed no significant differences between the subgroups.

**Figure 3:**
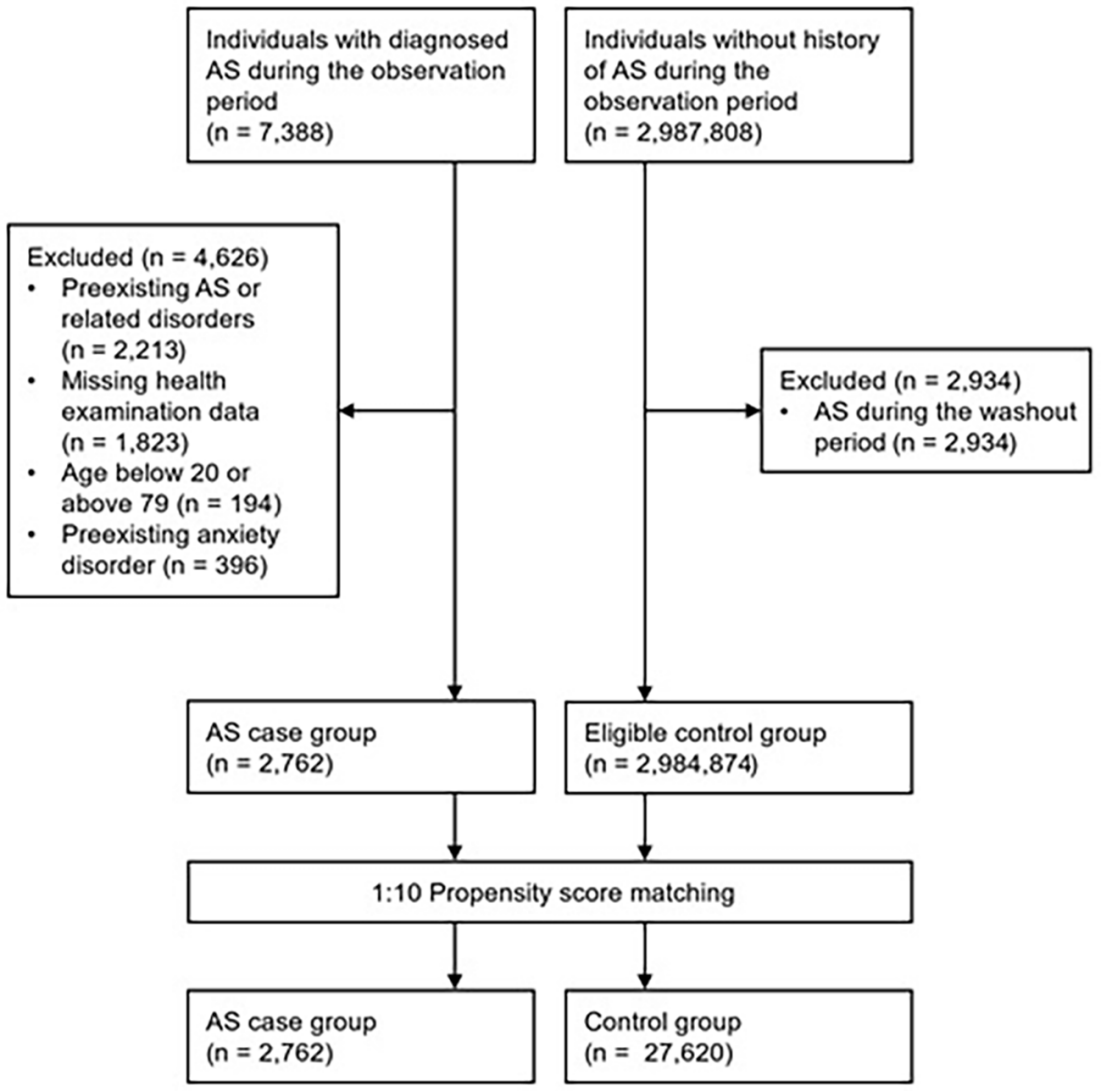
Adjusted hazard ratios for anxiety disorder following ankylosing spondylitis compared with values for matched controls stratified by age and sex. CI, confidence interval.

**Figure 3** presents the adjusted hazard ratios (aHRs) derived from the Cox proportional hazards model after adjusting for all baseline demographic characteristics. The AS cohort had an approximately 40% higher risk of anxiety disorders than the control cohort (aHR, 1.40; 95% CI, 1.14–1.73). In subgroup analyses by age and sex, the aHR was higher in younger individuals (<60 years; 1.56; 95% CI, 1.23–2.00) than in older individuals (≥60 years; 1.08; 95% CI, 0.73–1.60), with the latter not reaching statistical significance. The aHR was lower for males (1.30; 95% CI, 1.00–1.68) than for females (1.60; 95% CI, 1.13–2.26).

These findings indicate that AS is an independent risk factor for anxiety disorders, with the association being particularly pronounced in females aged < 60 years. This suggests that the development of anxiety disorders may be influenced not only by age or lifestyle factors but also by disease-specific characteristics and the disease burden associated with AS. Therefore, mental health screening and early intervention should be prioritized, particularly for younger female patients with AS.

## 4 Discussion

### 4.1 Summary of main findings

In this study, patients with AS had a significantly higher risk of anxiety disorders, approximately 1.40 times higher, compared with the control group without AS. The risk was notably higher in individuals under 60 years of age(approximately 1.56 times higher than in older individuals) and in females(approximately 1.60 times higher than in males). In contrast, sociodemographic variables, such as smoking status, alcohol consumption, BMI, total cholesterol level, and income level, were not significantly associated with an increased risk of anxiety disorders.

### 4.2 Comparison with previous studies

Large-scale population-based studies assessing the risk of anxiety disorders in patients with AS are scarce. A retrospective cohort study using Taiwan’s National Health Insurance database from 2000 to 2009 reported an aHR of 1.848 (95% CI, 1.369–2.494) for anxiety disorders in patients with AS compared with age- and sex-matched controls (7). The lower aHR observed in the present study may be attributed to the application of a longer washout period and propensity score matching to minimize confounding factors. Unlike the Taiwanese study, which excluded only patients with prior AS during the washout period, the present study excluded individuals with AS and those with major psychiatric comorbidities during this period and employed propensity score matching to reduce residual confounding, enabling a more rigorous comparison.

Several small studies have reported a high prevalence of anxiety symptoms in patients with AS. Studies conducted in Turkey and Morocco using the Hospital Anxiety and Depression Scale (HADS) found that 19.5% and 60% of patients with AS, respectively, had clinically significant anxiety (8, 16). In a single-center study from India, 38% of 100 patients with AS, assessed using the Generalized Anxiety Disorder-7 (GAD-7) scale, had significant anxiety symptoms (17). The increased risk of anxiety disorders identified in the present study is consistent with previous findings.

### 4.3 Pathophysiological mechanisms

The elevated risk of anxiety disorders following AS diagnosis is probably multifactorial and involves psychological, pain-related, inflammatory, and genetic factors. First, the psychological stress of receiving an AS diagnosis may increase susceptibility to anxiety disorders. AS is characterized by restricted spinal mobility, chronic pain, and fatigue, which can impair daily functioning and limit social activities, thereby exacerbating psychological distress (11, 18).

Second, persistent chronic pain may induce functional changes in central anxiety regulation circuits, facilitating the development of anxiety disorders (19). Chronic pain and anxiety share a bidirectional relationship (20), which can negatively impact both mental and physical health, as well as the overall quality of life (21).

Third, systemic inflammation may serve as an important biological pathway in anxiety pathogenesis (22). Elevated levels of inflammatory cytokines, such as C-reactive protein, interleukin (IL)-6, and tumor necrosis factor-α (TNF-α), in AS can influence the central nervous system by altering neurotransmitter metabolism (23), contributing to the pathophysiology of psychiatric disorders, including depression (24).

Fourth, genetic factors, particularly HLA-B27–associated immune pathways, may contribute to increased anxiety risk. Misfolding or overexpression of HLA-B27 can induce endoplasmic reticulum stress and activate the unfolded protein response, which stimulates the IL-23/IL-17 axis and promotes the secretion of proinflammatory cytokines such as TNF-α and IL-17 (25, 26). These cytokines may impair neurotransmitter regulation and activate the hypothalamic–pituitary–adrenal axis, thereby exacerbating anxiety symptoms (22, 27).

### 4.4 Age

The increased risk of anxiety disorders was significant only in patients aged < 60 years. A meta-analysis reported that a younger age in AS was associated with higher levels of psychological stress (28), and a single-center study from India found that a younger age at diagnosis was associated with more severe anxiety symptoms (17).

Early onset of a chronic disease, such as AS, can disrupt critical life-stage processes such as career development and social relationship building, intensifying the psychological burden. A German study involving 356 patients with inflammatory rheumatic diseases reported that psychiatric comorbidities, including depression and anxiety disorders, were closely associated with reduced work ability in AS, with productivity losses of approximately 12% in mild disease and up to 25% in moderate-to-severe disease (29). These findings emphasize the need for early detection and proactive intervention in working-age populations.

### 4.5 Sex

In sex-stratified analyses, females with AS had a higher risk of anxiety disorders than men with AS. This is consistent with evidence indicating greater overall vulnerability to anxiety disorders in females (30) and that rheumatic diseases exert a disproportionately adverse impact on females’ mental health (6). Such sex differences may be related to the tendency of females with inflammatory diseases to experience a lower quality of life (31). Female patients with AS are more likely to present with severe peripheral joint involvement, higher Bath Ankylosing Spondylitis Disease Activity Index scores, and a lower quality of life (32). Moreover, females often experience atypical or mild early symptoms, leading to significantly longer diagnostic delays than males (33), and such delays and diagnostic uncertainty may contribute to or worsen anxiety symptoms. These findings suggest that the combined effects of anxiety vulnerability, greater disease activity, reduced quality of life, and diagnostic delay may underlie the increased risk in females.

### 4.6 Smoking

This study found no significant association between smoking status and the risk of anxiety disorders. Some studies have suggested that smoking is associated with psychiatric disorders in patients with AS. A cross-sectional study involving 5,825 patients with AS reported a significant association between smoking and the occurrence of psychiatric disorders, suggesting that smoking cessation may serve as a potential strategy to prevent the development of anxiety and depressive symptoms in this population (34). In contrast, a large-scale genetics-based Mendelian randomization analysis found no significant causal association between smoking and the occurrence of depressive or anxiety disorders (35). These mixed findings suggest that the relationship between smoking and psychiatric disorders is influenced by multiple mediating and confounding factors.

### 4.7 Body mass index

BMI is considered an important determinant of clinical outcomes and quality of life in patients with AS. Previous studies have reported that overweight or obese patients with AS experience more severe symptoms and greater functional limitations than those with a normal BMI (36). However, the present study found no significant differences in anxiety disorder risk according to BMI. This suggests that the development of anxiety disorders may be influenced more by psychosocial factors, such as illness perception, emotional coping strategies, and social support, than by physical disease severity alone.

### 4.8 Income level

Although not statistically significant, the risk of anxiety disorders was approximately 1.6-fold higher in the low-income group and 1.4-fold higher in the high-income group, indicating a tendency toward a greater risk increase among those with lower income. This trend may reflect the disproportionate effect of AS-related physical limitations on individuals with fewer economic resources. Low-income patients may have reduced access to healthcare, rehabilitation services, and social support networks, which increases the likelihood that the physical burden of AS leads to psychological distress. A study of 866 patients with AS found that lower socioeconomic status was associated with greater disease severity and permanent disability, independent of biological DMARD use (37). Economic insecurity and job loss may further exacerbate psychological instability, contributing to increased anxiety risk. These findings highlight the need for mental health interventions personalized to the socioeconomic context in AS care.

### 4.9 Clinical implications

Anxiety in AS is not merely an emotional reaction but is also linked to central sensitization mechanisms (38), disease activity (10, 11), patient-reported pain intensity (8), and diminished quality of life (9, 16). Given that AS typically develops in younger adults and follows a chronic course, unmanaged anxiety symptoms can accumulate over time, adversely affecting long-term physical and mental health. Therefore, clinicians should incorporate active screening and early interventions for anxiety into routine AS management.

### 4.10 Study limitations and future directions

This study has several strengths, including the use of a large, nationally representative cohort with up to nine years of follow-up, application of a 3-year washout period and propensity score matching to minimize confounding, and stratified analyses by age and sex to identify high-risk groups. Nevertheless, several limitations should be acknowledged.

First, although distinguishing truly incident from long-standing undiagnosed AS is imperfect, the RID program’s strict diagnostic criteria, the requirement for repeated diagnostic coding, and the applied washout period collectively reduce but do not eliminate the likelihood of misclassification. Notably, while approximately 92% of patients with AS develop symptoms before the age of 45 years (39), nearly 25% of patients in the present cohort were aged ≥60 years at diagnosis. Second, the reliance on ICD-10 claims data precluded detailed information on treatment regimens, disease activity measures, and medication use. Unmeasured clinical variables may therefore have influenced our results: high disease activity could independently exacerbate psychological distress, certain treatments such as corticosteroids or biologics may induce psychiatric side effects, and genetic predispositions including HLA-B27 may contribute to systemic inflammation and neuroimmune pathways linked to anxiety. Third, important potential confounders, such as family psychiatric history, education level, physical activity, and medication history, were unavailable, leaving the possibility of residual confounding. Fourth, ascertainment bias cannot be excluded, as AS patients typically have more frequent medical visits, which may increase the likelihood of psychiatric coding. Although the washout period likely reduced this bias, and healthcare contact was further aligned by selecting controls from NHSP participants with recent screening records, residual bias remains possible. Finally, the outcome definition was deliberately restricted to ICD-10 codes F40–F41, which are DSM-5–concordant anxiety disorders coded by board-certified psychiatrists in Korea, in order to enhance diagnostic specificity.

Future research should incorporate clinical data on treatment exposures, disease activity indices, genetic information, and lifestyle factors to enable a more comprehensive evaluation of the association between AS and anxiety disorders. Such efforts would inform the development of integrated disease management strategies that address both physical and mental health outcomes in patients with AS.

## 5 Conclusion

In this nationwide population-based cohort study, patients with AS had a significantly increased risk of anxiety disorders, with particularly elevated risks among younger individuals and females. These findings underscore the need for early mental health screening and tailored interventions in high-risk AS populations to alleviate psychological burden and improve overall health outcomes.

## Data Availability

The dataset used in this study is from the Korean National Health Insurance Service and is not publicly available. Requests to access these datasets should be directed to Youngoh Bae, yobae05@gmail.com.
