# Supplementary material for: Risk of anxiety disorder following ankylosing spondylitis, 2012-2023: a nationwide cohort study in South Korea
Source: Front Psychiatry. 2026 Feb 13;17:1686890. doi: 10.3389/fpsyt.2026.1686890 (PMC12946093; doi:10.3389/fpsyt.2026.1686890)
Supplement: Supplementary file 1 [file Table1.docx]

Supplementary Material

**Table S1.** International Classification of Diseases, 10th Revision (ICD-10) codes used for the exclusion criteria in the study cohort selection

| **Disease** | **ICD-10 code** |
| --- | --- |
| Phobic anxiety disorders | F40 |
| Other anxiety disorders | F41 |
| Major psychiatric comorbidities | |
| Mood disorders | F30–F39 |
| Schizophrenia spectrum and other psychotic disorders | F20–F29 |
| Post-traumatic stress disorder (PTSD) and adjustment disorders | F43 |
| Obsessive–compulsive disorder | F42 |
| Reaction to severe stress, and adjustment disorders | F43, |
| Dissociative (conversion) disorders | F44 |
| Somatoform disorders | F45 |
| Major neurologic diseases | |
| Dementia (Alzheimer disease, frontotemporal dementia, etc.) | G30–G32 |
| Multiple sclerosis | G35 |
| Epilepsy and seizure disorders | G40–G41 |
| Cerebrovascular diseases (hemorrhage, infarction, sequelae) | I60–I69 |
| Head injury (particularly traumatic brain injury) | S06 |
| Fibromyalgia | M79.7 |
| Chronic Pain Syndrome | G89.4 |
| Other serious systemic diseases | |
| Malignant neoplasms  (especially brain, spinal cord, and hematologic malignancies) | C00–C97 |
| Systemic lupus erythematosus (SLE) | M32 |
| Rheumatoid arthritis (RA) | M05, M06 |
| Congenital malformations of the nervous system | Q00–Q07 |
| Congenital malformations of the circulatory system | Q20–Q28 |
| Chromosomal abnormalities | Q90–Q99 |
| Major inherited metabolic disorders | |
| Disorders of amino-acid metabolism (e.g., phenylketonuria) | E70–E72: |
| Lipid/carbohydrate metabolism disorders | E75–E77: |
| Other axial spondyloarthropathies / non-radiographic axSpA | |
| Other inflammatory spondylopathies | M46.x: |
| Sacroiliitis, not elsewhere classified | M46.0 |
| Other spondylitis | M46.1–M46.9 |
| Peripheral arthropathy–related conditions | |
| Psoriatic and enteropathic arthropathies | M07.x |
| Psoriatic arthritis mutilans | M07.0 |
| Polyarthritis | M07.1 |
| Spondylitis in psoriatic arthritis | M07.2 |
| Arthritis in Crohn’s disease | M07.3 |
| Arthritis in ulcerative colitis | M07.4 |
| Psoriasis (especially L40.5 arthropathic psoriasis) | L40.x |
| Inflammatory bowel disease (IBD) | |
| Crohn's disease | K50.x |
| Ulcerative colitis | K51.x |
